# Cytotoxicity of Cannabinoids in Combination with Traditional Lymphoma Chemotherapeutic Drugs Against Non-Hodgkin’s Lymphoma

**DOI:** 10.3390/biomedicines14010003

**Published:** 2025-12-19

**Authors:** Saba Omer, Mahmoud Mansour, Satyanarayana R Pondugula, Muralikrishnan Dhanasekaran, Brad Matz, Omer Khan, Dawn Boothe

**Affiliations:** 1 Department of Anatomy, Physiology, and Pharmacology, College of Veterinary Medicine, Auburn University, Auburn, AL 36830, USA; szo0027@auburn.edu (S.O.); srp0010@auburn.edu (S.R.P.); 2 Department of Drug Discovery and Development, Harrison School of Pharmacy, Auburn University, Auburn, AL 36849, USA; 3 Department of Clinical Sciences, College of Veterinary Medicine, Auburn University, Auburn, AL 36849, USA; 4 Samuel Ginn College of Engineering, Auburn University, Auburn, AL 36849, USA

**Keywords:** lymphoma, cannabinoids, CHOP, combination therapy, cancer

## Abstract

**Background**: Cannabinoids (CBs) are FDA-approved for mitigating chemotherapy-induced side effects such as pain, nausea, and loss of appetite. Beyond palliative care, CBs exhibit anti-tumor properties in various cancers, including non-Hodgkin’s lymphoma (NHL). Previously, we demonstrated the cytotoxic effect of endogenous and exogenous cannabinoids on human and canine B- and T-cell-type NHL cell lines. The purpose of this study was to establish the cytotoxic effect of cannabinoids in combination with the components of CHOP and lomustine. This traditional NHL chemotherapy regimen comprises cyclophosphamide, doxorubicin, vincristine, and prednisolone. **Methods**: In this study, we studied three cannabinoids, one from each of the three major categories of cannabinoids (endocannabinoid AEA, phytocannabinoid CBD, and synthetic cannabinoid WIN-55 212 22). Each cannabinoid was selected based on potency, as determined in our previous experiments. For the combination, we used five NHL chemotherapy drugs. We analyzed the cytotoxicity of each drug alone and in combinations using canine malignant B-type NHL cell line 1771 and a colorimetric MTT (3-(4,5-dimethylthiazol-2-yl)-2,5 diphenyl tetrazolium bromide) cell proliferation assay and combination index (CI) based on the Chou–Talalay method. **Results**: Our results demonstrate that the cytotoxic effects of all traditional NHL chemotherapy drugs are synergistically enhanced (interaction with CI < 1) by each of the three cannabinoids at sub-IC_50_ concentrations. **Conclusions**: This work provides a proof of concept for using cannabinoids and traditional NHL drugs in combination to reduce the dose, and thereby potentially reducing the toxicity, of chemotherapeutic drugs and increasing the survival benefit in lymphoma clinical translation studies, offering a significant advancement in cancer treatment.

## 1. Introduction

Cancer is one of the most common causes of death. As a significant global health concern, it is characterized by a persistently high incidence and mortality rate in both humans and canines [1]. Therapeutic options and chemotherapeutic regimens vary depending on the type of cancer. The majority of chemotherapeutic drugs target genes or proteins associated with cancer cell proliferation or survival pathways [2]. However, most of these drugs cause severe side effects due to their cytotoxicity on normal non-target cells. In addition, cancer cells also develop drug resistance through multiple mechanisms, including increased chemotherapeutic efflux [3]. As such, combination therapy has long been adopted as the standard first-line treatment of many malignancies to generate synergistic drug actions, deter the onset of drug resistance, and improve the clinical outcome [4,5,6]. Despite the benefit, some combinations increase the risk of host toxicity and while not resolving other factors contributing to therapeutic failure, such as drug resistance [7]. Therefore, a novel combination of chemotherapeutic drugs is required to address the current chemotherapy-related issues in several cancer models [8].

Canine lymphoma (CL) is a common type of neoplasia in dogs, with an estimated incidence rate of 20–100 cases per 100,000 dogs and is in many respects comparable to non-Hodgkin’s lymphoma in humans. Canine and human lymphoma are generally characterized by a high rate of initial remission following conventional CHOP (cyclophosphamide, (hydroxy) doxorubicin, vincristine, and prednisolone)-based therapies, providing a reassuring sign of the effectiveness of current treatments. However, 95% of dogs and 30% of humans will succumb to drug-resistant relapse [9,10]. In humans, several newer therapeutic modalities—such as targeted agents including Bruton tyrosine kinase (BTK) inhibitors (e.g., ibrutinib) [11], monoclonal antibodies and antibody–drug conjugates (e.g., polatuzumab vedotin, loncastuximab) [12], bispecific T-cell engagers (e.g., mosunetuzumab, glofitamab) [13], and CD19-directed CAR-T-cell therapies (e.g., axicabtagene ciloleucel, tisagenlecleucel) [14]—have significantly improved outcomes for subsets of patients with relapsed or refractory NHL. However, these advances have not been translated to canine lymphoma, in which such therapies remain unavailable, financially impractical, or insufficiently validated [15,16,17]. This translational gap underscores the ongoing need for innovative therapeutic approaches for drug-resistant lymphoma in both species.

Over the past two decades, cannabinoids have been increasingly studied for their anticancer properties and have demonstrated inhibitory effects on tumor growth, survival, and metastasis across several malignancies [18,19,20]. Mechanistically, cannabinoids modulate several pathways implicated in chemoresistance, including activation of intrinsic apoptotic signaling via caspase-3/9, suppression of PI3K/AKT-mediated survival signaling, inhibition of MAPK/ERK phosphorylation, and reduction in P-glycoprotein expression—thereby enhancing intracellular retention of chemotherapeutic drugs [21]. In our previous studies, we have also demonstrated significant anticancer effects of endogenous and exogenous cannabinoids in canine and human NHL cell lines [22,23].

Given that traditional NHL chemotherapeutic drugs exert their cytotoxic effects partly through induction of apoptosis and disruption of survival pathways, targeting overlapping mechanisms with cannabinoids provides a strong rationale for exploring potential synergistic interactions.

In this study, we set out to analyze the potential of cannabinoids in combination with traditional lymphoma chemotherapeutic drugs as a novel treatment strategy for canine B-cell lymphoma. By leveraging complementary mechanisms of action, this approach aims to provide a more effective and potentially less toxic therapeutic option for drug-resistant lymphoma.

## 2. Materials and Methods

### 2.1. Reagents and Chemicals

A Thiazolyl Blue Tetrazolium Bromide (MTT) assay for cell proliferation was purchased from American Type Culture Collection. Penicillin–streptomycin solution was purchased from Thermofisher Scientific (Waltham, MA, USA). Cell culture RPMI 1640 Medium, ES Cell Qualified fetal bovine serum (FBS) and L-glutamine solution were purchased from EMD Millipore (Burlington, MA, USA). Phosphate-buffered saline (PBS) and dimethylsulfoxide (DMSO) were purchased from Sigma Aldrich (St. Louis, MO, USA). All the cannabinoids (AEA, CBD, and WIN, Table 1) and chemotherapeutic drugs (DOX, VIN, PRD, and LOM, Table 1) were purchased from Sigma Aldrich (St. Louis, MO, USA).

### 2.2. Cell Maintenance

An authenticated canine B-cell lymphoma cell line 1771 was generously shared by Dr. Steven Suter’s Lab (North Carolina State University) (RRID: CVCL_0B18). All cells were cultured in RPMI 1640 Medium, supplemented with fetal bovine serum (10%), penicillin–streptomycin (1%), and L-glutamine (1%). Lymphoma suspension cells were grown and harvested via centrifuge for the cell viability assay. Cells were then seeded into 96-well plates at a density of 1 × 10^4^ cells/well. Cells were incubated under standard conditions at 37 °C and supplemented with 5% CO_2_.

### 2.3. IC_50_ Calculation

To calculate the IC_50_ of each cannabinoid and chemotherapeutic drug, each drug was dissolved in its respective vehicle and then further dissolved in the media to the desired concentrations. Cells were treated with the individual drug for 24 or 48 h, depending on the drug. For each dose–response curve, eight replicates were performed per concentration, and the entire experiment was repeated three independent times to ensure reproducibility. Dose–response data were processed using SAS (version 9.4) and GraphPad Prism^®^ (version 9.5.1). IC_50_ values were obtained by applying linear regression analysis to the log-transformed dose–response data. The IC_50_ value for each drug was then calculated using the following formula.

IC_50_ = 50 − *Intercept estimate*/*Concentration estimate*

The intercept estimate and concentration estimate were calculated using linear regression analysis [24] in the SAS statistical package [25].

### 2.4. Drug Combination

All drugs were dissolved in their respective vehicle and further diluted in RPMI-1640 media to the desired concentrations. Drug mixtures for the calculation of CI values were based on the median-effect analysis method [26]. Two-fold serial dilutions of working concentrations were prepared in RPMI-1640 with three concentrations above and three concentrations below the calculated IC_50_ for each drug in each cell line. These ratios corresponded to 0.12, 0.25, 0.5, 1, 2, 4, and 8 times the IC_50_ for each drug. Cannabinoid/chemotherapeutic drug mixtures were made using those ratios for each drug. Each concentration was tested in eight replicates for individual drugs and combinations, and the results were confirmed in at least three independent experiments. Randomization was used to prepare the combinations and assign them to wells. The investigator remained blinded to the drug identity during cell treatment and data analysis.

### 2.5. Cell Viability

The effect of the drug combinations on the viability of cells was determined by (3-[4,5-dimethylthiazol-2-yl]-2,5-diphenyl tetrazolium bromide) (MTT) assay. The cells were plated at 1 × 10^4^ cells per well in 100 µL of complete culture medium containing the appropriate drugs for 24 or 48 h (depending on the drug) at 37 °C in a humidified chamber. After incubation for specified times at 37 °C in a humidified incubator, MTT reagent (10 µL) was added to each well and incubated for 4 h, followed by adding 100 µL of solubilization solution per well to dissolve formazan crystals. Color absorbance (OD) was recorded on an Appliskan^®^ microplate reader (Thermo Fisher Scientific, Vantaa, Finland) at a 570 nm wavelength. The effect of drugs on cell viability was assessed as the percentage of inhibition in cell viability, where vehicle-treated cells were taken as 100% viable. Each experiment was repeated three times.

### 2.6. Data Analyses

Data from MTT viability assays were expressed as the mean ± SD. GraphPad Prism^®^ (version 9.5.1) was used to analyze the MTT data and produce dose–response curves for cannabinoids and chemotherapeutic drugs using nonlinear analysis. To calculate the drug effect, the mean OD values for each drug concentration were subtracted from the mean OD values of cells treated with vehicle only. The resulting fractions (between 0–100%) were plotted against drug concentrations on a logarithmic scale.

Combination index (CI) values were determined by the third-generation “CompuSyn” software written by Nick Mart of MIT using the median-effect method [27], derived from the mass-action law principle, which is the unified theory that provides the common link between single entities and multiple entities and first-order and higher-order dynamics. This general equation encompasses the Michaelis–Menten, Hill, Henderson–Hasselbalch, and Scatchard equations in biochemistry and biophysics [26].

CI = CA,X/ICX,A + CB,X/ICX,B

where CA, X and CB, X are the concentrations of cannabinoids and chemotherapeutic drugs used in combination to achieve X% drug effect. ICX, A and ICX, B are the concentrations for single drugs (cannabinoids or chemotherapeutic) that achieve the same effect. The sum of CA, X/ICX, A and CB, X/ICX, B is defined as the combination index at effect X, as indicated by the CI equation above. Synergy is defined as CI < 1, additivity is defined as CI = 1, and antagonism is defined as CI > 1.

## 3. Results

### 3.1. Determination of IC_50_ for Cannabinoids and NLC Drugs in 1771 Lymphoma Cells

**Figure 1:**
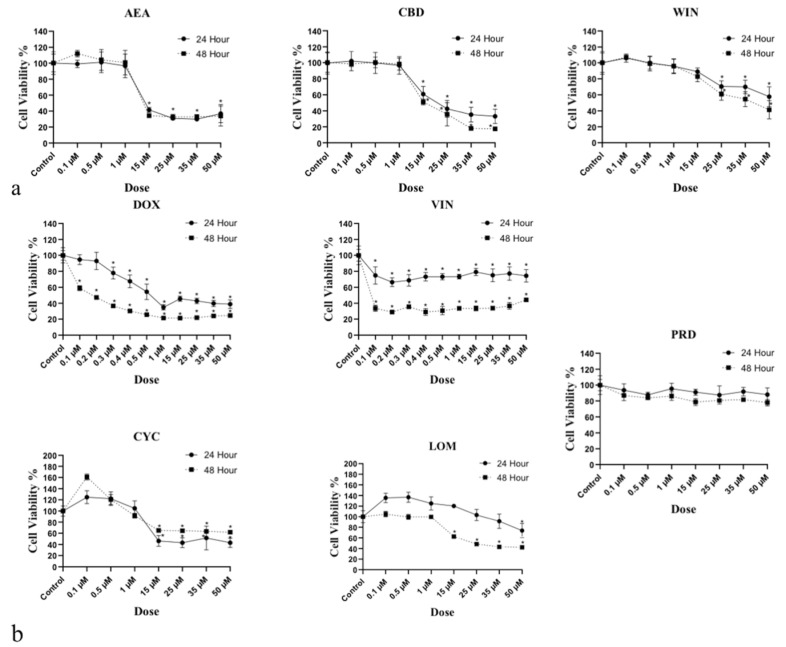
IC_50_ of cannabinoids (AEA, CBD, and WIN) and NLC drugs (DOX, CYC, VIN, LOM, and PRD): (**a**) calculation of IC_50_ for cannabinoids (AEA, CBD, and WIN) using MTT dose–response curves expressed as the drug concentration vs. viability/proliferation of 1771 lymphoma cells. CB IC_50_s were 14, 23, and 69 μM for AEA, CBD, and WIN, respectively, in 1771 cells; (**b**) calculation of IC_50_ for NLC drugs (DOX, CYC, VIN, LOM, and PRD) using MTT dose–response curves expressed as the drug concentration vs. viability/proliferation of 1771 lymphoma cells. NLC drug IC_50_s were 0.73, 31, 0.25, 27, and 44 μM for DOX, CYC, VIN, LOM, and PRD, respectively, in 1771 cells. * *p* > 0.005.

To identify the cannabinoid–NLC drug interactions as synergism, additive effects, or antagonism, we calculated the dose–effect curves for cannabinoids and NLC drugs applied singly to canine B-cell lymphoma cells. This step generated the IC_50_—the drug concentration causing 50% cell growth inhibition for each drug—that is requisite for the CI calculation. In the drug combination studies, the combination index (CI) indicates the following: CI < 1 indicates synergism, CI = 1 indicates an additive effect, and CI > 1 indicates antagonism. Two-fold serial dilutions of each drug were used in the in vitro MTT experiments, and data showed that the CB or NLC treatments inhibited the proliferation of 1771 cells with variable IC_50_ values. CB IC_50_s were 14, 23, and 69 μM for AEA, CBD, and WIN, respectively (Figure 1). NLC drug IC_50_ values were 0.73, 31, 0.25, 27, and 44 µM, respectively, for DOX, VIN, CYC, LOM, and PRD (Figure 1). IC_50_ values fell within the range commonly reported in cancer cell studies [28,29,30].

### 3.2. Combination of CBs and NLC Drugs Synergistically Caused the Death of 1771 B Lymphoma Cells

**Figure 2:**
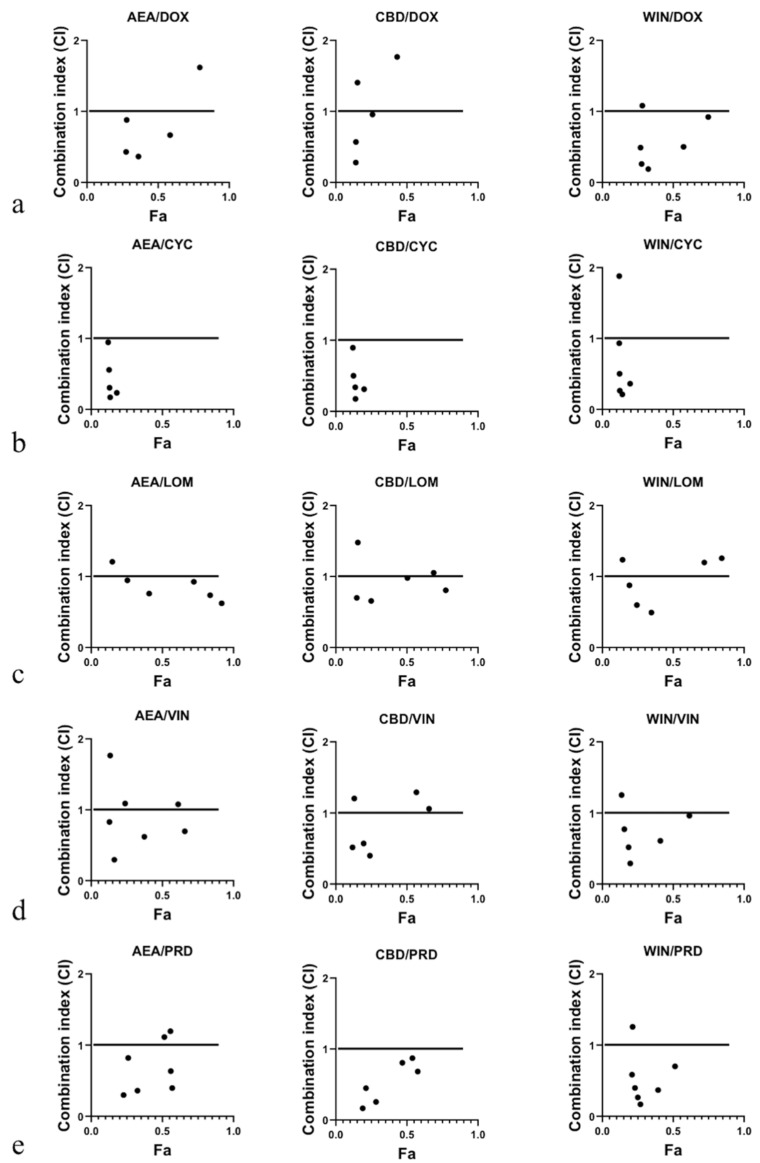
Combination index (CI) analysis of cannabinoid (CB) interactions with NLC drugs in 1771 lymphoma cells. CI values derived from dose–effect MTT assays are shown for each CB–drug combination (CI < 1: synergism; CI = 1: additivity; CI > 1: antagonism). (**a**) AEADOX, CBDDOX, and WINDOX plots show predominantly synergistic interactions with DOX. (**b**) AEACYC, CBDCYC, and WINCYC combinations demonstrated synergism at higher fractional effects (Fa > 0.5). (**c**) LOM combinations (AEALOM, CBDLOM, and WINLOM) showed both synergistic and antagonistic effects. (**d**) CB combinations with VIN (AEAVIN, CBDVIN, and WINVIN) also exhibited mixed interactions, including synergistic, additive, and antagonistic points. (**e**) PRD combinations (AEAPRD, CBDPRD, and WINPRD) were largely synergistic. Note: Seven data points were used for each drug combination. However, few CI values were simulated beyond the plotting domain. These data points were included in the analysis but have limited therapeutic relevance.

To determine whether CBs influence drug-induced cytotoxicity in 1771 lymphoma cells in a synergistic, additive, or antagonistic manner, dose–effect MTT assays were performed. Analysis using isobolograms [31] and combination index (CI) calculations revealed CI values < 1 for all drug combinations, indicating overall synergistic interactions. However, several synergistic data points were observed at higher fractional effects (Fa > 0.5), which carry less therapeutic relevance. In particular, combinations of CBs with CYC showed synergism only at Fa > 0.5 (Table 2 and Figure 2). Overall, these findings indicate that at lower doses, combining CBs with NLC drugs leads to significantly greater inhibition of cell growth compared with treatment using individual agents.

- Combination index plots for CBs + DOX:
  The AEADOX plot includes five data points, with four showing synergism (CI < 1) and one showing antagonism (CI > 1). CBDDOX similarly presents five data points, three being synergistic and two antagonistic. The WINDOX plot contains six data points, five demonstrating synergism and one showing an additive effect.
- Combination index plots for CBs + CYC:
  Both AEACYC and CBDCYC plots contain five data points, all demonstrating synergism (CI < 1), though all are at Fa > 0.5. The WINCYC plot shows six data points, five being synergistic and one antagonistic, again all simulated at Fa > 0.5.
- Combination index plots for CBs + VIN:
  The AEAVIN plot includes seven data points: four synergistic, two additive, and one antagonistic. CBDVIN presents six data points, with three showing synergism, one being additive, and two being antagonistic. The WINVIN plot includes six data points, five being synergistic and one antagonistic.
- Combination index plots for CBs + LOM:
  The AEALOM plot contains six data points, five being synergistic and one antagonistic, with the antagonistic point occurring at a low Fa value. CBDLOM shows six data points, four being synergistic, one being additive, and one being antagonistic. The WINLOM plot includes six data points, with three being synergistic and three antagonistic.
- Combination index plots for CBs + PRD:
  The AEAPRD plot includes seven data points, five being synergistic and two additive. CBDPRD presents six data points, all being synergistic (CI < 1). The WINPRD plot includes seven data points, with six demonstrating synergism and one being antagonistic.

### 3.3. CBs Combined with NLC Drugs at IC_50_ Synergistically Inhibited 1771 B-Cell Lymphoma Cell Growth

We conducted cell viability assays on 1771 cells using the IC_50_ concentration of each drug, tested either alone or in combination with CBs.

- CBs + DOX at IC_50_:
  CBD significantly potentiated DOX-induced inhibition of 1771 cell growth, demonstrating a synergistic effect at IC_50_. In contrast, AEA and WIN did not show synergistic activity with DOX at this concentration.
- CBs + CYC at IC_50_:
  No significant potentiating or synergistic effect of any CB on CYC-induced cytotoxicity was observed when the drugs were combined at their IC_50_ concentrations.
- CBs + VIN at IC_50_:
  All three CBs—AEA, CBD, and WIN—significantly enhanced VIN-induced inhibition of 1771 cell viability, indicating a synergistic effect at IC_50_.
- CBs + LOM at IC_50_:
  AEA, CBD, and WIN each significantly potentiated LOM-induced cytotoxicity in 1771 cells when combined at IC_50_.
- CBs + PRD at IC_50_:
  All three CBs significantly increased PRD-induced inhibition of 1771 cell growth at IC_50_.

Results are expressed as percentage change relative to the control (Mean ± SD).

**Figure 3:**
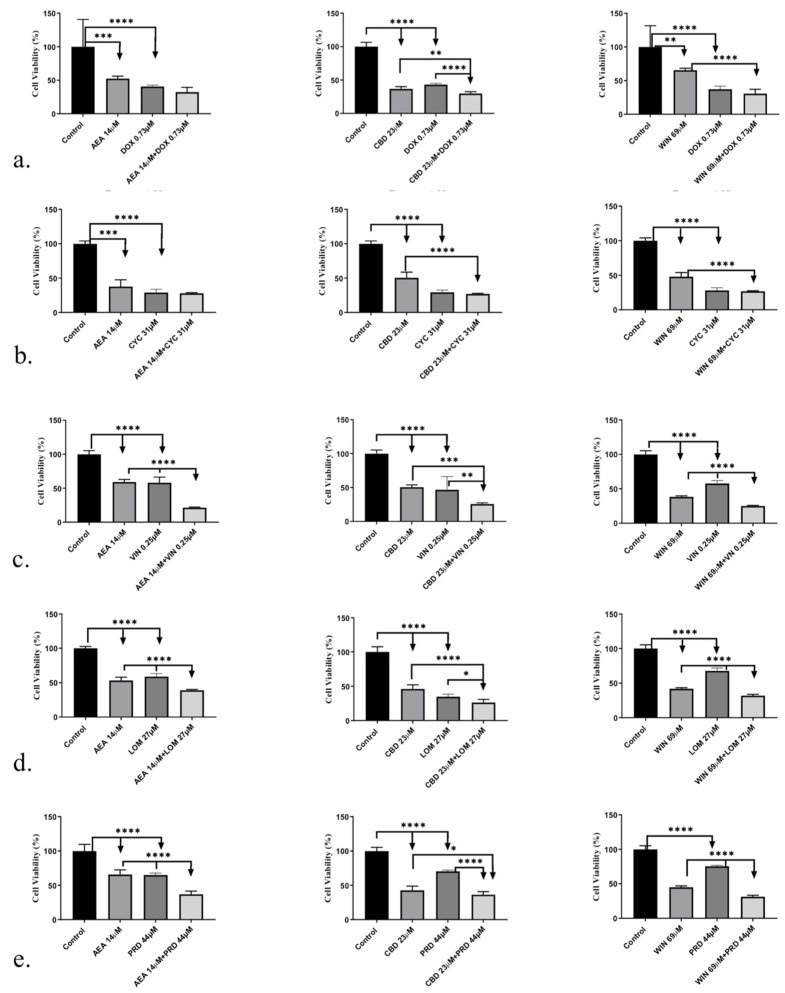
Effect of CBs combined with NLC drugs on 1771 lymphoma cell viability at IC_50_. (**a**) CBD significantly potentiated DOX-induced inhibition of 1771 cell growth, whereas AEA and WIN showed no synergistic effect at IC_50_. (**b**) No significant potentiation of CYC-induced cytotoxicity was observed with any CB at IC_50_. (**c**) AEA, CBD, and WIN significantly enhanced VIN-induced inhibition of 1771 cell growth. (**d**) All three CBs significantly potentiated LOM-induced inhibition at IC_50_. (**e**) AEA, CBD, and WIN significantly increased PRD-induced inhibition at IC_50_. Results are presented as percentage change relative to the control (Mean ± SD). Statistical significance was determined by one-way ANOVA followed by appropriate post hoc testing. * *p* < 0.05, ** *p* < 0.01, *** *p* < 0.001, **** *p* < 0.0001.

Overall, treatment with the IC_50_ concentration of individual CBs or NLC drugs produced approximately 50% cell death, whereas combining CBs with several NLC drugs at their IC_50_ concentrations led to a significant increase in cytotoxicity (Figure 3a,c,d,e). However, no significant synergistic effect was observed for any CB combined with CYC, nor AEA combined with DOX, at IC_50_ (Figure 3b).

## 4. Discussion

This study demonstrated that combining CBs with traditional lymphoma chemotherapeutic drugs can significantly enhance their anticancer effects, particularly in 1771 lymphoma cells. This finding not only opens new possibilities in cancer treatment but also underscores the potential of our work to make a significant contribution to the field.

We treated 1771 canine lymphoma cells with CBs and NLC drugs, singly or in combination, to document the effect of these drugs on lymphoma cells. The isobologram and CI analysis showed that the CI for CB combination with NLC drugs was <1 or =1 in low doses (0.12×, 0.25×, and 0.5× IC_50_), indicating synergistic and additive relationships, respectively. However, at higher doses (2×–8× IC_50_), we also found an antagonistic relation (CI > 1) of the combinations, likely due to dose-related factors such as receptor desensitization, pathway saturation, or stress-induced cellular responses that diminish therapeutic interaction [19,32,33]. Also, in case of prednisolone, particularly its combination with AEA (Table 3), synergy was observed only at moderate effect levels (ED_50_/IC_50_), but higher effect levels revealed strong antagonism, which limits the therapeutic potential of these combinations, particularly AEA/PRD. These findings highlight that the beneficial synergy between cannabinoids and NLC drugs is dose-dependent and most effective within sub-IC_50_ ranges.

We also observed synergistic and antagonistic simulations at Fa < 1. These simulations indicate that the combinations are less relevant therapeutically since cancer cell death in small numbers is considered less valuable in treating cancer. This understanding is crucial for the development of effective cancer treatments. To further validate the results of our CI analysis, we analyzed the viability of 1771 cells treated with IC_50_ concentrations of each CB and NLC drug individually. We compared the result with the viability of cells treated with the combination at IC_50_ concentration and untreated control cells. Our results demonstrated the potentiated effect of CBs on NLC drugs’ cytotoxicity. However, at IC_50_ concentrations, we could not demonstrate the synergistic effect of AEA on DOX and any cannabinoid used in this study on cyclophosphamide-induced inhibition of 1771 cell growth.

The IC_50_ values observed for AEA, CBD, and WIN (14–69 μM) fall within the micromolar range commonly reported for cannabinoids in cancer cell models and are considered pharmacologically plausible given their lipophilicity and ability to accumulate in tissues [29,30]. In contrast, the IC_50_ values obtained for the NLC drugs are consistent with published cytotoxicity profiles of CHOP components [34] reflecting known differences in activation requirements, intracellular uptake, and mechanisms of action. These observations help contextualize the relative potency of the tested agents and support the biological relevance of evaluating their combined effects in canine B-cell lymphoma.

Gustafsson et al. (2009) studied the effect of endogenous and synthetic CBs in combination with the chemotherapeutic drug 5-fluorouracil in colorectal carcinoma cells. Their study demonstrated the synergistic effect of synthetic cannabinoids on the chemotherapeutic drug but not with endocannabinoid AEA [35]. Our results of the IC_50_ combination of CBD with CYC are similar to those of Andradas et al. (2021) in vitro study, in which they analyzed the effect of CBs in combination with cyclophosphamide in medulloblastoma cells [36]. Further, Strong et al. (2018) showed the synergistic effect of cannabidiol with other traditional lymphoma chemotherapeutic drugs, such as ibrutinib, and proteasome inhibitors, such as carfilzomib [31,37].

A limitation of this study is that all experiments were conducted using a single canine B-cell lymphoma cell line (1771). Combination index assays require extensive dose–response matrices, so we focused on one well-characterized model to complete the large number of required combinations. However, in our previous studies, we have evaluated cannabinoid responses across multiple canine and human lymphoma cell lines and observed consistent trends. Building on that foundation, this study provides a focused proof of concept for drug–cannabinoid interactions. Future work will extend these combination analyses to additional lymphoma cell lines and primary samples to improve generalizability.

## 5. Conclusions

In summary, the findings from this study show that a combination of CBs and traditional lymphoma chemotherapeutic agents produces a dose-dependent, synergistic increase in 1771 lymphoma cell death at sub-IC_50_ concentrations. This discovery opens the potential for reducing the dose necessary to inhibit cancer cell growth and, thus, the nonspecific toxicity associated with it by incorporating cannabinoids. In line with the results of our previous studies, the synergistic effect of CBs with lymphoma chemotherapeutic drugs is a further indication of the potential benefits of targeting the cannabinoid pathway for treating malignant lymphoma.

## Figures and Tables

**Table 1:** Chemical structures and abbreviations of cannabinoids and chemotherapeutic drugs used.

Cannabinoids (CBs) Panel	NHL Chemotherapy Drugs Panel (NLC)
Anandamide (AEA)—Endocannabinoid 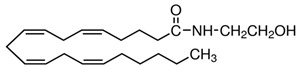	Doxorubicin (DOX) 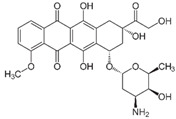
	Cyclophosphamide (CYC) 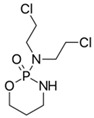
Cannabidiol (CBD)—Phytocannabinoid 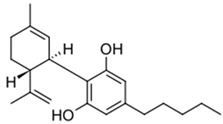	Lomustine (LOM) 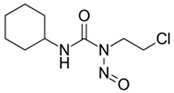
	Vincristine (VIN) 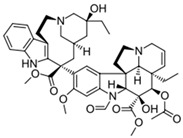
WIN 55-212 22 (WIN)—Synthetic Cannabinoid 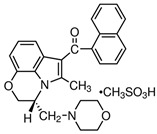	Prednisolone (PRD) 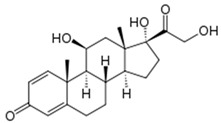

**Table 2:** Combination index (CI) values.

Drug Combinations	CI Values at Corresponding Doses						
	0.25	0.5	1	2	4	8	16
AEA/DOX	1.61992	0.66614	0.36450	0.42845	0.87930	2.02629	5.70658
CBD/DOX	1.76727	2.57811	0.95555	0.27889	0.56966	1.40582	12.9357
WIN/DOX	0.92142	0.50069	0.18778	0.25971	0.48934	1.08157	2.66036
AEA/CYC	0.23358	0.1704	0.30561	0.55785	0.94597	2.31856	4.33812
CBD/CYC	0.3133	0.17504	0.33841	0.50016	0.89482	2.00064	12.3526
WIN/CYC	0.36316	0.2129	0.26403	0.50293	0.93256	1.8816	2.77754
AEA/VIN	0.69691	1.07749	0.62071	0.29664	1.0893	0.829	1.76577
CBD/VIN	1.05909	1.29037	0.40006	0.57188	0.51804	1.20373	4.38346
WIN/VIN	0.96306	0.60887	0.28864	0.51851	0.77303	1.25329	2.03859
AEA/LOM	0.62206	0.73607	0.92653	0.75897	0.94603	1.20716	2.4846
CBD/LOM	0.80517	1.05218	0.97975	0.65612	0.6996	1.47968	5.22987
WIN/LOM	1.25745	1.19752	0.49299	0.59787	0.87586	1.23573	2.37321
AEA/PRD	0.3966	0.63436	1.1947	1.11198	0.35895	0.2973	0.81838
CBD/PRD	0.34493	0.46539	0.48758	0.31461	0.44162	0.92014	2.60304
WIN/PRD	0.70135	0.36793	0.16719	0.26345	0.39777	0.58383	1.25507

Note: [CI < 1 indicates synergism; CI > 1 indicates antagonism; and CI = 1 indicates additive effect. CI is obtained by dividing CBs IC_50_/NLC drugs IC_50_].

**Table 3:** Combination index (CI) at 50–95% effect.

	Combination Index (CI) at 50–95% Effect			
Drug Combination	ED50	ED75	ED90	ED95
AEA/DOX	0.78232	0.34562	0.17554	0.11614
CBD/DOX	0.62255	0.28710	0.13356	0.07974
WIN/DOX	0.45266	0.21730	0.10565	0.06518
AEA/CYC	<0.001	<0.001	<0.001	<0.001
CBD/CYC	<0.001	<0.001	<0.001	<0.001
WIN/CYC	<0.001	<0.001	<0.001	<0.001
AEA/VIN	0.66737	0.52721	0.41795	0.35757
CBD/VIN	0.66684	0.43908	0.28932	0.21794
WIN/VIN	0.46094	0.28730	0.18033	0.13186
AEA/LOM	0.98415	0.75361	0.57993	0.48661
CBD/LOM	0.92988	0.58054	0.36441	0.26628
WIN/LOM	0.92655	0.78793	0.67335	0.60671
AEA/PRD	0.41611	34.6162	10311.2	497964.
CBD/PRD	0.46768	0.89025	4.48310	14.9966
WIN/PRD	0.26785	0.59590	6.96326	42.1908

Note: [CI < 1 indicates synergism; CI > 1 indicates antagonism; and CI = 1 indicates additive effect. CI is obtained by dividing CBs IC50/NLC drugs IC50], ED = effective dose.

## Data Availability

The data presented in this study are available on request from the corresponding author.

## Abbreviations

The following abbreviations are used in this manuscript:

CBs	Cannabinoids
NHL	Non-Hodgkin’s lymphoma
CHOP	Traditional chemotherapeutic regimen for NHL
AEA	Linear dichroism
CBD	Cannabidiol
WIN	WIN-55 212 22
DOX	Doxorubicin
CYC	Cyclophosphamide
LOM	Lomustine
VIN	Vincristine
PRD	Prednisolone
IC_50_	Half-maximal inhibitory concentration
CI	Combination index
MTT	Thiazolyl blue tetrazolium bromide

## References

[1] Meng J., Guo F., Xu H., Liang W., Wang C., Yang X.-D. (2016). Combination therapy using co-encapsulated resveratrol and paclitaxel in liposomes for drug resistance reversal in breast cancer cells in vivo. Sci. Rep..

[2] Galustian C., Dalgleish A.G. (2010). Article Commentary: The power of the web in cancer drug discovery and clinical trial design: Research without a laboratory?. Cancer Inform..

[3] Lehne G. (2000). P-glycoprotein as a drug target in the treatment of multidrug resistant cancer. Curr. Drug Targets.

[4] Lavi O., Gottesman M.M., Levy D. (2012). The dynamics of drug resistance: A mathematical perspective. Drug Resist. Updates.

[5] Hu C.-M.J., Zhang L. (2012). Nanoparticle-based combination therapy toward overcoming drug resistance in cancer. Biochem. Pharmacol..

[6] Hiss D.C., Gabriels G.A., Folb P.I. (2007). Combination of tunicamycin with anticancer drugs synergistically enhances their toxicity in multidrug-resistant human ovarian cystadenocarcinoma cells. Cancer Cell Int..

[7] Iwamoto T. (2013). Clinical application of drug delivery systems in cancer chemotherapy: Review of the efficacy and side effects of approved drugs. Biol. Pharm. Bull..

[8] Kummar S., Chen H.X., Wright J., Holbeck S., Millin M.D., Tomaszewski J., Zweibel J., Collins J., Doroshow J.H. (2010). Utilizing targeted cancer therapeutic agents in combination: Novel approaches and urgent requirements. Nat. Rev. Drug Discov..

[9] Hosoya K., Kisseberth W.C., Lord L.K., Alvarez F.J., Lara-Garcia A., Kosarek C.E., London C.A., Couto C.G. (2007). Comparison of COAP and UW-19 protocols for dogs with multicentric lymphoma. J. Vet. Intern. Med..

[10] Harrison J.S., DesMarteau P., Hamilton B., Patthoff S. (2012). Leukemia and Lymphoma Society.

[11] Mouhssine S., Maher N., Matti B.F., Alwan A.F., Gaidano G. (2024). Targeting BTK in B cell malignancies: From mode of action to resistance mechanisms. Int. J. Mol. Sci..

[12] Sehn L.H., Herrera A.F., Flowers C.R., Kamdar M.K., McMillan A., Hertzberg M., Assouline S., Kim T.M., Kim W.S., Ozcan M. (2020). Polatuzumab vedotin in relapsed or refractory diffuse large B-cell lymphoma. J. Clin. Oncol..

[13] Budde L.E., Sehn L.H., Matasar M., Schuster S.J., Assouline S., Giri P., Kuruvilla J., Canales M., Dietrich S., Fay K. (2022). Safety and efficacy of mosunetuzumab, a bispecific antibody, in patients with relapsed or refractory follicular lymphoma: A single-arm, multicentre, phase 2 study. Lancet Oncol..

[14] Neelapu S.S., Locke F.L., Bartlett N.L., Lekakis L.J., Miklos D.B., Jacobson C.A., Braunschweig I., Oluwole O.O., Siddiqi T., Lin Y. (2017). Axicabtagene ciloleucel CAR T-cell therapy in refractory large B-cell lymphoma. N. Engl. J. Med..

[15] Impellizeri J.A., Howell K., McKeever K.P., Crow S.E. (2006). The role of rituximab in the treatment of canine lymphoma: An ex vivo evaluation. Vet. J..

[16] Zandvliet M. (2016). Canine lymphoma: A review. Vet. Q..

[17] Majchrzak A., Witkowska M., Mędra A., Zwolińska M., Bogusz J., Cebula-Obrzut B., Darzynkiewicz Z., Robak T., Smolewski P. (2013). In vitro cytotoxicity of ranpirnase (onconase) in combination with components of R-CHOP regimen against diffuse large B cell lymphoma (DLBCL) cell line. Adv. Hyg. Exp. Med./Postep. Hig. Med. Dosw..

[18] Guzman M. (2003). Cannabinoids: Potential anticancer agents. Nat. Rev. Cancer.

[19] Hinz B., Ramer R. (2019). Anti-tumour actions of cannabinoids. Br. J. Pharmacol..

[20] Ramer R., Hinz B. (2017). Cannabinoids as anticancer drugs. Adv. Pharmacol..

[21] Holland M.L., Panetta J.A., Hoskins J.M., Bebawy M., Roufogalis B.D., Allen J.D., Arnold J.C. (2006). The effects of cannabinoids on P-glycoprotein transport and expression in multidrug resistant cells. Biochem. Pharmacol..

[22] Omer S., Pathak S., Nadar R., Bowen D., Sandey M., Dhanasekaran M., Pondugula S., Mansour M., Boothe D. (2023). Validating the anti-lymphoma pharmacodynamic actions of the endocannabinoids on canine non-Hodgkin lymphoma. Life Sci..

[23] Omer S., Pathak S., Mansour M., Nadar R., Bowen D., Dhanasekaran M., Pondugula S.R., Boothe D. (2024). Effects of cannabidiol, ∆9-tetrahydrocannabinol, and WIN 55-212-22 on the viability of canine and human non-Hodgkin lymphoma cell lines. Biomolecules.

[24] Ramsey F., Schafer D. (2012). The Statistical Sleuth: A Course in Methods of Data Analysis.

[25] Rodriguez R.N. (2011). Sas. Wiley Interdiscip. Rev. Comput. Stat..

[26] Chou T.-C. (2010). Drug combination studies and their synergy quantification using the Chou-Talalay method. Cancer Res..

[27] Chou T.-C., Talalay P. (1984). Quantitative analysis of dose-effect relationships: The combined effects of multiple drugs or enzyme inhibitors. Adv. Enzym. Regul..

[28] Velasco G., Hernández-Tiedra S., Dávila D., Lorente M. (2016). The use of cannabinoids as anticancer agents. Prog. Neuropsychopharmacol. Biol. Psychiatry.

[29] Millar S.A., Stone N.L., Bellman Z.D., Yates A.S., England T.J., O’Sullivan S.E. (2019). A systematic review of cannabidiol dosing in clinical populations. Br. J. Clin. Pharmacol..

[30] Ohlsson A., Lindgren J.E., Andersson S., Agurell S., Gillespie H., Hollister L.E. (1986). Single-dose kinetics of deuterium-labelled cannabidiol in man after smoking and intravenous administration. Biomed. Env. Mass. Spectrom..

[31] Huang R.-y., Pei L., Liu Q., Chen S., Dou H., Shu G., Yuan Z.-x., Lin J., Peng G., Zhang W. (2019). Isobologram analysis: A comprehensive review of methodology and current research. Front. Pharmacol..

[32] Howlett A.C., Blume L.C., Dalton G.D. (2010). CB(1) cannabinoid receptors and their associated proteins. Curr. Med. Chem..

[33] Atalay S., Jarocka-Karpowicz I., Skrzydlewska E. (2019). Antioxidative and anti-inflammatory properties of cannabidiol. Antioxidants.

[34] He J., Hajj K.A., Knapp C.M., Whitehead K.A. (2019). Development of a clinically relevant chemoresistant mantle cell lymphoma cell culture model. Exp. Biol. Med..

[35] Gustafsson S.B., Lindgren T., Jonsson M., Jacobsson S.O. (2009). Cannabinoid receptor-independent cytotoxic effects of cannabinoids in human colorectal carcinoma cells: Synergism with 5-fluorouracil. Cancer Chemother. Pharmacol..

[36] Andradas C., Byrne J., Kuchibhotla M., Ancliffe M., Jones A.C., Carline B., Hii H., Truong A., Storer L.C., Ritzmann T.A. (2021). Assessment of cannabidiol and Δ9-tetrahydrocannabiol in mouse models of medulloblastoma and ependymoma. Cancers.

[37] Strong T., Rauvolfova J., Jackson E., Pham L.V., Bryant J. (2018). Synergistic effect of cannabidiol with conventional chemotherapy treatment. Blood.

